# Does Increasing Farm Plot Size Influence the Visual Quality of Everyday Agricultural Landscapes?

**DOI:** 10.3390/ijerph20010687

**Published:** 2022-12-30

**Authors:** Kristina Janeckova Molnarova, Iris C. Bohnet, Kamila Svobodova, Kateřina Černý Pixová, Michael Daniels, Jan Skaloš, Kristýna Drhlíková, Hossein Azadi, Roman Zámečník, Petr Sklenička

**Affiliations:** 1 Department of Landscape and Urban Planning, Faculty of Environmental Sciences, Czech University of Life Sciences Prague, Kamýcká 129, 165 00 Praha-Suchdol, Czech Republic; 2 Department of Agricultural Economics and Rural Development, University of Göttingen, 37073 Göttingen, Germany; 3 Department of Geography and the Environment, University of Denver, Denver, CO 80210, USA; 4 Department of Applied Ecology, Faculty of Environmental Sciences, Czech University of Life Sciences Prague, Kamýcká 129, 165 00 Praha-Suchdol, Czech Republic; 5 Department of Economics and Rural Development, Gembloux Agro-Bio Tech, University of Liège, 5030 Gembloux, Belgium; 6 Faculty of Environmental Science and Engineering, Babeș-Bolyai University, 400000 Cluj-Napoca, Romania; 7 Department of Planting Design and Maintenance, Faculty of Horticulture, Mendel University in Brno, Valtická 337, 691 44 Lednice, Czech Republic

**Keywords:** landscape aesthetics, spatial heterogeneity, agricultural landscape, land systems, land-use policy

## Abstract

The increase in farm plot size is one of the most apparent and significant trends that have influenced central and eastern European agricultural landscapes since the 1950s. In many countries where the average plot size in traditional land-use systems did not exceed several hectares, present-day plots reach the size of 200 ha or more. In recent times, efforts have been made to reverse this trend to restore important ecosystem functions and to re-establish the aesthetic values of everyday landscapes. Visual landscape quality is becoming a major driving force in the development of agricultural landscapes with known effects on people’s well-being and health, and this quality plays an increasingly important role in agricultural policies. However, no comprehensive research has been carried out to establish the links between perceived visual landscape quality and the scale of the farm plot pattern. The current study was therefore designed to determine whether greater farmland pattern heterogeneity, i.e., smaller farm plot sizes, is consistent with higher visual preferences. The results showed that people preferred a small-scale plot pattern in landscapes characterized by a flat relief and a low proportion of woody vegetation. These homogeneous landscapes were also overall considered significantly less beautiful than more diverse landscapes. However, even a moderate decrease in plot size notably improved these low beauty scores. These preferences were displayed consistently by all respondents, and most strongly by older respondents, respondents with a higher level of education, and those professionally engaged in landscape design or conservation. The high level of consensus among respondents in rejecting further land consolidation in homogeneous landscapes, which form a large proportion of European farmland, underlines that the results of this study provide a valid argument for discussing sustainable agricultural plot sizes as part of agricultural policy-making.

## 1. Introduction

Agricultural landscapes have traditionally fulfilled multiple functions, serving not only as a resource for the production of food and industrial crops, but also as the home and workplace of most of the human population, and as a habitat for wildlife and plants [1,2]. However, in recent decades, the role of agricultural landscapes has been evolving at an increasing pace [3,4]. On one hand, this landscape is, comparatively speaking, depopulating, as an increasing proportion of people live and work in towns and cities [5,6]. Intensification of agriculture has also led to a significant loss in the habitat and conduit functions of these landscapes [7,8]. On the other hand, the rural countryside is increasingly utilized for outdoor recreation [9,10,11]. Where agricultural landscapes are located close to towns and cities, they become the everyday landscape for large numbers of urbanites and exurbanites [12,13], who project their paradigms and dreams into these landscapes [3] and become a major force in the shaping of these rural areas [14,15]. As a result, there has been an increasing focus on preserving the ecological [16,17,18] and cultural [19,20] values of these landscapes, initiated by their new users [21]. 

While traditional landscape preservation has been associated with “landscape mummification” [22], there is now a shared view that land-use planning should focus on managing the future landscape rather than simply protecting the past fabric [23], pursuing a balance between preservation and development. The current problems of the agricultural landscape are clarified by the relaxation of the harmonious relationship between community–territory–economy and cultural practices that influenced the historical landscape. In particular, focusing attention on agricultural landscapes has become relevant in those territories with a special vocation for agro-livestock [24]. Leaving aside the different definitions of agricultural landscape that can be given [25], there has been a need to understand agricultural landscape changes and the links between the changes and the policies adopted. Being aware of the changes that are taking place allows us to understand and properly manage the trends that are underway or, if necessary, to address them in the future. 

Some studies have explored the impact of changes in demand for agricultural products and the effect of agricultural production on landscape quality from a macroeconomic point of view, focusing on changes in the agricultural sector, or from a local point of view, by examining changes in landscape for small case studies [17,18,26]. It is a rather complex field of research to determine the value of agricultural land [25], which has been studied for a long time by different authors and is still the subject of research and analysis. Variations in land values are undoubtedly a manifestation of land appreciation on the market; given their dynamics, it may be useful to understand which areas are most vulnerable to possible land-use changes [18]. Although various studies have addressed different points of view, the agricultural landscape theme has yet to be seen as a key economic aspect [26,27]. 

Specifically in central and eastern Europe, one of the most apparent and significant trends that have influenced agricultural landscapes in recent decades is the increase in the average size of the farm plots, i.e., area of farmland that is managed as a homogenous unit in terms of farm operations and cultivated crop [28,29]. This trend, aimed at improving agricultural production, took place mainly from the 1950s [30,31,32]. Thus, the diversity of landscape structures has dramatically declined across the countryside, leading to the homogenization of landscapes [33,34,35,36,37]. In many of the central and eastern European countries, traditional land-use systems called pluzinas or Hufenflur were in place for centuries, with plot size not exceeding several hectares [38]. As a result of socialist land-use policies and the collectivization process, consolidation of arable plots took place, followed by the increase in the mean size of arable land plots [30]. The process was ultimately undertaken in conjunction with a campaign to industrialize agriculture, which had previously been based on traditional methods. High productivity, high inputs of pesticides and fertilizers, low proportion of permanent grasslands in the landscape [33], and excessive mechanization and plot size have characterized intensive agriculture [39]. Since heavy agricultural machinery could be used more efficiently and could produce larger crops on larger plots compared with numerous small farms on small plots, plot size has increased rapidly [40,41]. In recent years, selected CAP greening measures aim to promote higher crop diversity in the farming landscapes, leading to a decrease in farm plot sizes [42] and increased functional landscape heterogeneity: cf. [17]. However, the above mentioned impacts of landscape development in the second half of the 20th century largely prevail [43].

This change in landscape structure has been the focus of research in a number of disciplines, especially within the environmental sciences. Researchers [31,44] have outlined the concept of land-use fragmentation, which reflects the pattern of the landscape seen by bird’s eye, i.e., areas of the farming landscapes occupied by individual crops. A landscape that is lacking in field boundaries stabilized by woody vegetation and that is at the same time homogenous from the standpoint of land-use fragmentation has been shown to be more prone to water and tillage erosion [45,46] and also increased surface runoff and muddy floods [47,48]. Studies addressing the change in landscape structure from a biodiversity and wildlife conservation perspective emphasize the importance of non-productive patches in the agricultural landscape [49,50,51,52], as well as spatial and temporal diversity in functional cover types, represented by high land-use fragmentation [17,53]. By contrast, the concept of land ownership fragmentation describes the pattern of the fields defined by their ownership as depicted by the Land Cadastre. Sklenicka et al. [31] have illustrated that, perhaps counter-intuitively, high land ownership fragmentation often leads to large fields used for one crop and all associative negative impacts on the landscape.

In current landscape planning practices, indicators adopted from landscape ecology are often used to measure or describe visual landscape quality [54], often without specific knowledge concerning the visual relevance of these concepts [55]. However, visual landscape quality is becoming a major driving force in the development of agricultural landscapes [16,56], and this quality plays an increasingly important role in agricultural policies [57,58,59]. Specific research exploring visual preferences of the broad public for agricultural landscapes has therefore been attracting increased attention. Many empirical studies have risen to this challenge, and a majority of them have concentrated on the major influence of individual features forming the agricultural landscape [60,61]. It has been established that the perception of agricultural landscapes is strongly influenced by landscape vegetation [62,63,64,65], water elements [66,67,68], or meadows [69]. Among studies focusing on the overall features of a landscape, Clay and Smidt [70], as well as Lovell and Sullivan [71], noted that vividness, variety, and unity are generally regarded as the most influential features in this respect, while other authors also emphasized openness [72,73] and color contrast [74]. Many studies [75,76,77] have also indicated a relationship between typical agrarian landscape attributes and aesthetic values of landscape, while Scott [78] and Daugstad et al. [79] have demonstrated a preference for traditional farming practices in the countryside. However, to our knowledge, no study has so far focused directly on the visual impact of plot size and the resulting diversity of farmland patterns. 

This study, therefore, aims to determine the effect of farmland pattern heterogeneity on visual preferences for various types of everyday agricultural landscapes, i.e., agricultural landscapes that are not recognized or protected for outstanding scenic value. We aim to verify the following hypotheses: (1) Respondents attribute higher aesthetic value to agricultural landscapes with smaller farm plots than to otherwise identical landscapes with larger farm plots. (2) Respondent’s evaluation of landscape aesthetic quality in relation to farm plot size will further depend on respondents’ sociodemographic characteristics. 

## 2. Materials and Methods

### 2.1. Agricultural Landscape Types

**Figure 1:**
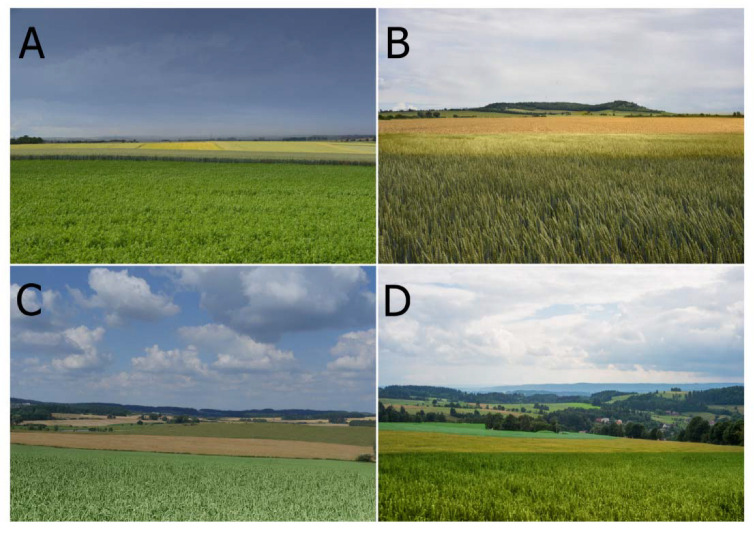
Four different types of agricultural landscapes (**A**–**D**) were used in our research. (**A**)—the lowest baseline landscape heterogeneity, determined by flat terrain and almost no three-dimensional landscape structures; (**B**)—second lowest baseline landscape heterogeneity, determined by flat terrain with a forested horizon; (**C**)—higher baseline landscape heterogeneity, determined by a slightly rolling morphology, a forested horizon and medium-dense vegetation structures; (**D**)—highest baseline landscape heterogeneity, determined by a distinctive morphology and dense vegetation structures.

This study investigated four types of agricultural landscapes with different baseline landscape heterogeneity (Figure 1). For each of the landscape types, a total of 100 photographs of everyday agricultural landscapes were taken in the agrarian regions of the Czech Republic. The photographs were taken using a digital camera with a basic focal length of 50 mm and a portable tripod at a height of 170 cm, i.e., from the average adult’s view. The landscapes were photographed from roads commonly used in the regions. Based on a consensus of authors, four representative photographs were selected to use in the following quantitative analyses. 

Baseline landscape heterogeneity was determined by vectorizing the photographs and calculation of the percentage of woody vegetation elements (i.e., trees, woodlands, and hedgerows, cf. [80]) in each landscape. In the calculation, the proportional occurrence of each feature was used as it appears in the photograph, as this reflected the people’s perception of the landscape more accurately [81]. The proportion of woody vegetation in photograph A was 1.4%, for photograph B it was 3.0%, for photograph C it was 6.8%, and for photograph D it was 14.9%, confirming the increasing diversity of these landscapes.

The first two landscapes (A and B) contain few distinctive geomorphological and three-dimensional landscape features, while the other two (C and D) are more heterogeneous. Landscape A represents agricultural landscapes often found in fertile lowland areas, with a flat relief and almost no visible three-dimensional vegetation structures. Landscape B is similar to landscape A but has a distinctive and forested horizon. Landscape C has a slightly rolling morphology, with a forested horizon and some visible vegetation structures, including solitary trees, small wood patches, and hedgerows, mostly in the background. Landscape D is characterized by a relatively distinctive morphology and a complex vegetation pattern including solitary trees, wood patches of various sizes, hedgerows, and tree lines. 

**Figure 2:**
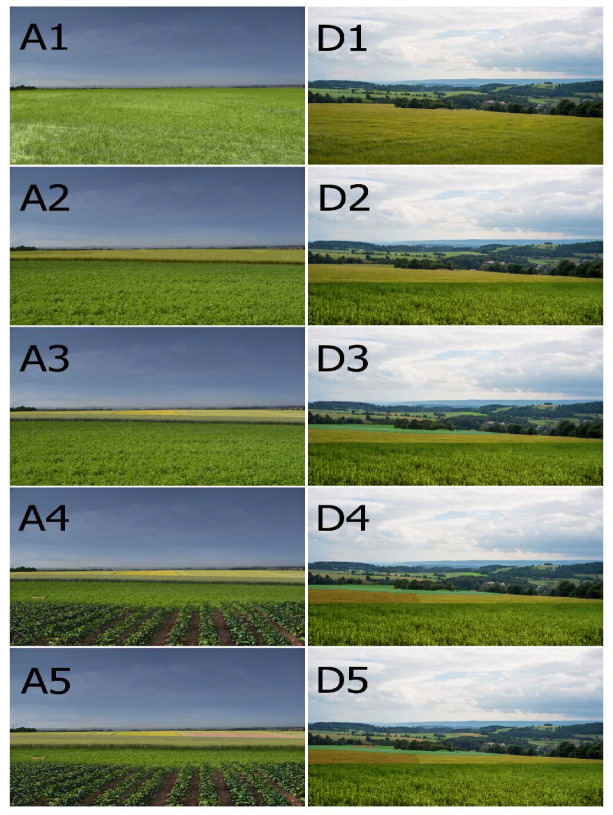
Landscapes A and D represent the landscape types with the lowest and highest baseline landscape heterogeneity, respectively. Each landscape is depicted in a series of five images according to the manipulated plot size, illustrated by a variety of crops with different colors and shapes. Plots of approximately 50 ha in size are part of landscapes A1 and D1, followed by plots of about 35 ha in landscapes A2 and D2, 20 ha plots in landscapes A3 and D3, 10 ha plots in landscapes A4 and D4, and 5 ha plots in landscapes A5 and D5.

Each of the four photographs was subsequently manipulated in the Adobe Photoshop program to create a series of five photographs of the same landscapes but with varying plot sizes (i.e., varying land-use fragmentation), illustrated by a diversity of crops with different colors and shapes. The series included the original photograph. This photo-manipulation resulted in a set of 20 images with different plot sizes representing 4 agricultural landscape types. The landscapes of the largest plot size contained plots of approximately 50 hectares (see Figure 2, landscape A1 and landscape D1), followed by landscapes containing plots of 35 ha, 20 ha, and 10 ha, respectively. The smallest plot size was represented by plots of 5 ha, as shown in Figure 2, landscape A5 and landscape D5, respectively. 

### 2.2. The Questionnaire Survey

To analyze the effect of farmland pattern heterogeneity on visual preferences for various types of agricultural landscapes, we conducted an online questionnaire survey. The questionnaire was designed in two core parts. The first part focused on visual preferences for the landscapes with varied plot size, while the second part collected data on respondents’ sociodemographic characteristics. Twenty images were used in the online questionnaire, together with seven control images without plot size manipulation. These photographs were taken in similar agricultural landscapes as the four photographs A-D and depicted areas that ranged from very little woody landscape vegetation and almost flat terrain (similar to landscape A) to areas with relatively abundant woody landscape vegetation and undulating terrain (similar to landscape D). The control images were included to ascertain that respondents did not react primarily to the graphic manipulation of the studied photographs. As respondents were not told that the evaluated landscapes represented a scale of baseline heterogeneity and plot size, the control images also served to make the sample more varied and the pattern less apparent. Each respondent was asked to evaluate two sets of images, where each set depicted five increasingly fragmented versions of the same landscape as shown in Figure 2, along with the seven control photographs. All images were presented in random order. Respondents were asked to evaluate the depicted landscapes based on their aesthetic preferences using a 7-point Likert-type scale ranging from 1 (very ugly) to 7 (very beautiful). 

The second part of the questionnaire recorded basic characteristics of the respondents: gender, age, education, place of birth, current place of residence, and their field of occupation or study. The characteristics “place of birth” and “current place of residence” were used to determine the region of the respondents’ birth and residence among the 14 administrative regions of the Czech Republic, the size of the municipalities of the respondents’ birth and residence in 5 categories (1–1000 inhabitants, 1001–10,000, 10,001–100,000, 1,000,001–1,000,000, and over 1,000,001), and whether the respondents had moved between regions or between municipalities of different size categories (Table 1). 

The survey took approximately 15 min and was undertaken in 2016. The collection of data was conducted in cooperation with a professional agency, which used its databases to address respondents forming a random balanced sample of the Czech population. Respondents were contacted by e-mail and telephone and filled in the questionnaire individually online. 

The random sample included 1033 respondents. This included 502 men (48.6%) and 531 women (51.4%) who were 18 years old or older. Respondents under 18 years old were not included in the study for legal reasons. The age distribution of the sample also reflects the demographic characteristic of the Czech nation, with the largest group of respondents being 36–52 years old (32.6%), as did the education structure, with the highest proportion of respondents with completed high school education (67.0%), followed by university-level education (22.1%). The largest group of respondents (30.5%) lived in municipalities of between 1000 and 10,000 inhabitants (30.2%), followed by people from large towns of 10,000 inhabitants or more (20.7%). 

### 2.3. Statistical Analysis of the Data 

A statistical evaluation was performed using IBM SPSS, v. 20.0. Description statistics were calculated for all characteristics and evaluated categories. Count and frequency were counted for categorical data (i.e., gender, age group, education, profession or study focus, place of origin, and place of residence). Mean, valid N, and Standard Deviation (SD) were used for the evaluated categories. Combinations of individual characteristics were tested using chi-square tests to reveal biases of the data; no significant bias was found. 

One-way ANOVA was used for comparing the respondents’ evaluation of the photographs with different levels of landscape heterogeneity (5–50 ha). 

ANOVA Repeated Measures (RM ANOVA) was applied, using the following parameters: WS factor: polynomial; SS type (III); homogeneity tests—descriptive; post hoc tests: Scheffe, Games–Howel; adj: Bonferoni; main factors and diversity degree; baseline p/significance level 0.05; and p/SL under 0.1 was partly taken into account. The Tukey criterion was used for evaluating individual differences between each pair of these photographs. 

Repeated Measures ANOVA is generally used for evaluation of time impact (i.e., no. of the visit in pharmaceutical studies). As evaluated photos were not standing alone (but as a group of five corresponding photos of the same origin but with increasing grade of diversity), it was necessary to compare and process the evaluation within the group and to measure the influence of diversity within one group. As landscape heterogeneity could be recognized as an ordinal variable, RM ANOVA was recognized as one of the few available ways how to statistically evaluate the influence of farm plot size on visual preferences expressed by the respondents.

## 3. Results

### Respondents’ Preferences for Different Landscapes and Farm Plot Size

**Figure 3:**
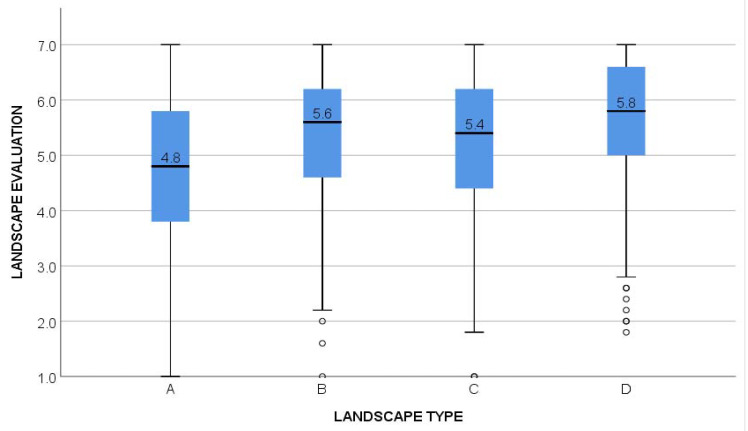
Average evaluation of each landscape A–D as assessed by respondents, who were asked to evaluate the depicted landscapes based on their aesthetic preferences using a 7-point Likert-type scale ranging from 1 (very ugly) to 7 (very beautiful).

The results showed that the difference in the respondents’ evaluation of the individual landscapes A–D was highly significant (*p* < 0.001; Figure 3). Overall, landscapes with a higher baseline heterogeneity were preferred over less diverse ones. Respondents rated landscape A1 with the lowest average score (4.49 out of a total of 7). Landscapes D2 and D3 got the highest average score (5.69 out of a total of 7). In the sample of the seven control photographs, the variance was higher, with scores ranging from 4.81 to 6.05. 

**Figure 4:**
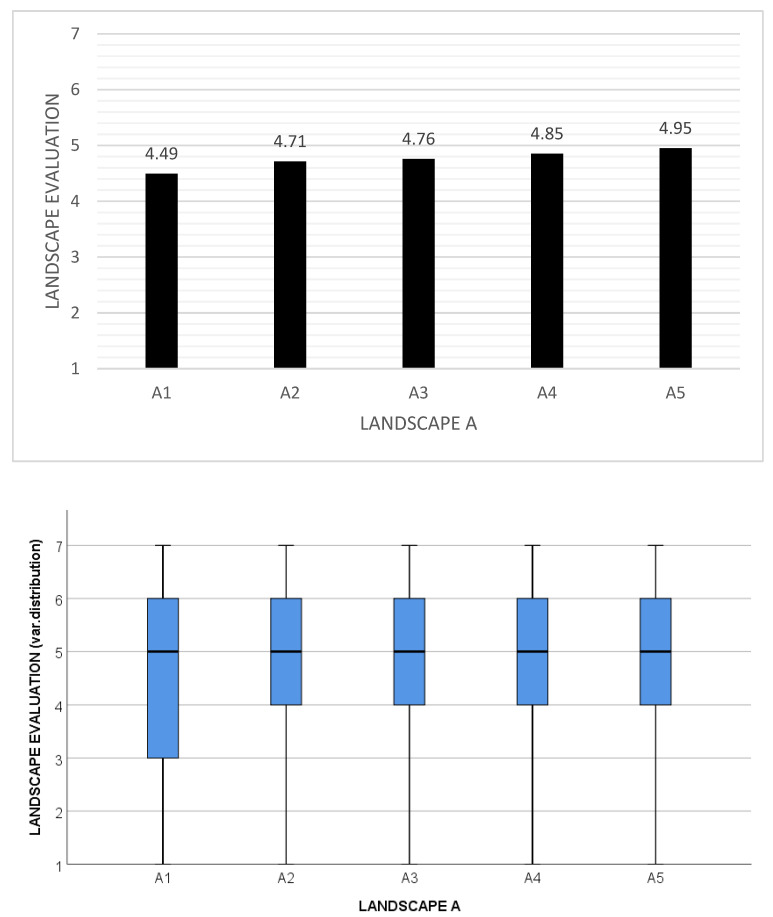

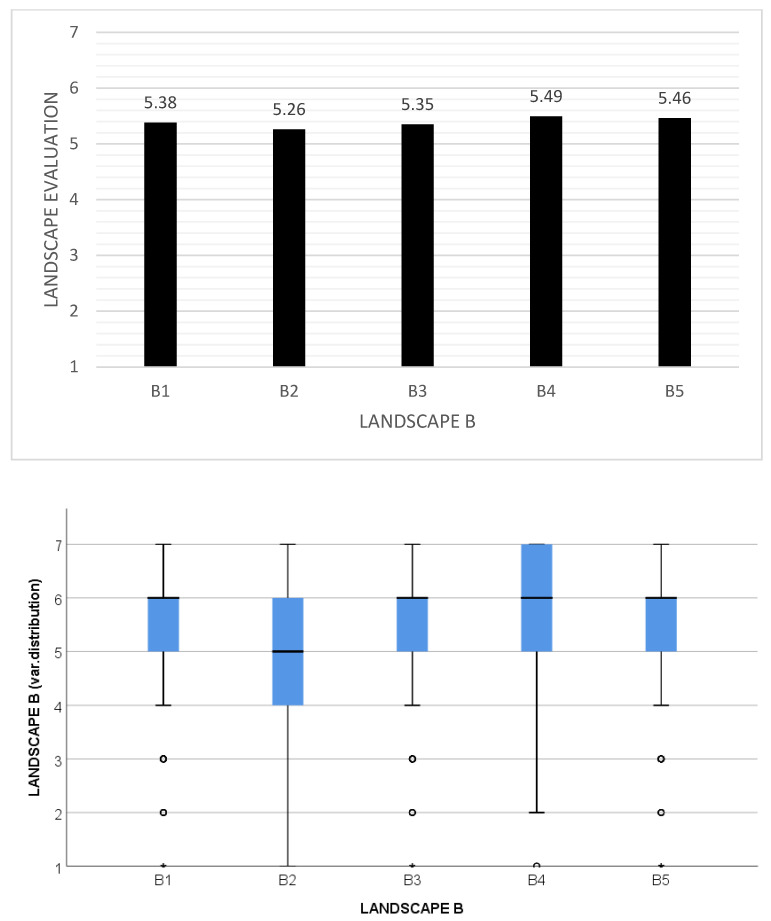
Evaluation of landscape types A and B (top—average values, bottom—variable distribution) with varying plot sizes A1–A5 and B1–B5, based on a 7-point Likert-type scale ranging from 1 (very ugly) to 7 (very beautiful). In both these landscape types with low baseline landscape heterogeneity, we found a significant preference for smaller agricultural plots (*p* = 0.003 for landscape A and *p* < 0.001 for landscape B).

The significant effect of agricultural plot size on respondents’ preferences for studied landscapes was confirmed in the landscape types A and B with the two lowest levels of baseline heterogeneity. We found a significant preference for smaller farm plots across all five variants of landscapes A and B (A1–A5 and B1–B5) (Figure 4).

In landscape A, there is a significant preference (*p* = 0.003) for smaller agricultural plots. Landscape A1 with the largest agricultural plots of approximately 50 ha received an overall score of 4.49, the lowest in the study, including the control photographs. However, landscape A5, the same landscape type but with the smallest plot size of approximately 5 ha, received a score of 4.95, which is higher than three of the seven control photographs. In landscape A, this link is manifested universally across the gender, age, education, and profession of the respondents. However, there are differences in the level and significance of preference for A landscapes between these groups. Overall, women ranked all five photographs higher than men (*p* = 0.002). There were also significant differences in the evaluation of these photographs by respondents with different levels of education (*p* = 0.029), with the exception of the most consolidated landscape (A1), which received an extremely low ranking from respondents across the education structure. Respondents with a university education evaluated landscapes A2–A5 as less beautiful compared with less educated respondents (*p* (A2) = 0.032; *p* (A3) = 0.001; *p* (A4) = 0.014; *p* (A5) = 0.036). 

We identified a stronger tendency to prefer a less consolidated landscape among university and high school educated respondents compared with respondents with primary education. However, this tendency did not prove statistically significant. With increasing age, the respondents were more critical toward this landscape (*p* < 0.001), and respondents above 52 years of age also manifested a stronger preference for small-scale landscapes (*p* < 0.001). Respondents in professions connected with landscape design and conservation (Group 1) were significantly more critical toward this landscape compared with respondents in other professions (Groups 2 and 3).

Landscape A is also the only landscape where the respondents’ preferences differed according to their region of origin. Respondents from the South-Moravian Region preferred this landscape (in all variants) significantly less than respondents from the Moravian–Silesian Region (*p* = 0.001). People who currently live in a municipality of the same size as the municipality where they were born liked all variants of this landscape significantly more (*p* = 0.008) than people who had moved to a smaller or larger municipality. People who had moved between regions during their lifetime awarded a higher rating for beauty to landscapes A3 (*p* = 0.047) and A5 (*p* = 0.048) compared with people who had stayed in the same region. 

In landscape B, a significant tendency toward the preference for a small-scale landscape was identified (*p* < 0.001, Figure 4). Respondents showed the highest preference for the small-scale landscape B4 (average score 5.49), while the highly consolidated landscape B2 received the lowest average score (5.26). This pattern of preference was manifested universally across respondents’ gender, age, education, and profession, but there were some differences in the level and significance of preference for small-scale landscapes between these groups.

Women ranked all five images higher than men (*p* = 0.006). The evaluation of this landscape by the respondents also differed significantly according to their age. The older the respondents were, the more critical they were of this landscape, and in variants B1–B3, this pattern of preference was statistically significant (*p* < 0.001). Respondents over 70 years of age also showed a higher preference for small-scale landscapes compared with other groups of respondents (*p* = 0.019). The evaluation of this landscape also varied significantly according to the profession of the respondent (*p* = 0.035). Especially respondents in professions connected with landscape design and conservation (Group 1, *p* < 0.001) and respondents working in farming and forestry (Group 2, *p* = 0.012), liked landscape B significantly less than respondents from the residual group (Group 3). 

The consolidated variants of this landscape, B1 and B2, were almost the only landscapes in the study where respondents’ opinions differed according to the size of their municipality. Landscape B1 was significantly more preferred by respondents who were born in a medium-size town of 10,001 to 100,000 inhabitants compared with respondents coming from large towns with more than 100,000 inhabitants and those coming from smaller municipalities with up to 10,000 inhabitants (*p* = 0.018). Landscape B2 was significantly more preferred by people who lived in medium-size towns of 10,001 to 100,000 inhabitants compared with respondents living in other municipalities (*p* = 0.009). 

In landscapes C and D, we found no significant differences in respondents’ evaluations according to farm plot size. Landscape C (Figure 1, average score 5.31) has a slightly rolling morphology, with a forested horizon and some landscape vegetation, mostly in the background. All variants of this landscape with various farm plot sizes received almost the same scores from the respondents. Respondents over 52 years of age evaluated all variants of this landscape as less beautiful (*p* < 0.001) compared with younger respondents, and respondents with a university education evaluated the two more consolidated variants of this landscape as less beautiful (*p* (C1) = 0.025, *p* (C2) = 0.031) compared with less educated respondents. Landscape D (Figure 1, average score 5.65) has a rolling morphology, a forested horizon, and several woody patches and hedgerows. This landscape received the highest overall ranking from the four tested landscapes, and the ranking was uniformly high for all variants of this landscape. This landscape was more highly evaluated by women than by men (*p* = 0.0095), while the variant with a medium level of consolidation (D3) was evaluated less favorably by respondents with a university education compared with other respondents (*p* = 0.03). 

To conclude, Hypothesis 1 (“Respondents attribute higher aesthetic value to agricultural landscapes with smaller farm plots than to otherwise identical landscapes with larger farm plots.”) was verified for landscapes A and B with lower baseline landscape heterogeneity, whereas in landscapes with higher baseline heterogeneity (C,D), visual preference was predominantly determined by factors other than plot size. Hypothesis 2 (“Respondent’s evaluation of landscape aesthetic quality in relation to farm plot size will further depend on respondents’ sociodemographic characteristics.”) has not been confirmed, as preference for smaller plots in otherwise homogeneous landscapes were manifested consistently across all respondent groups and throughout the Czech Republic. There were several exceptions to this general conclusion, which are noted below.

## 4. Discussion and Conclusions

The positive impact of smaller farm plot size on visual preferences was significant in landscapes A and B, i.e., the two landscapes with the lowest baseline landscape heterogeneity. In these landscapes, the smaller size of the farm plots creates a smaller scale, and thus brings diversity into an otherwise monotonous landscape. The landscape gains in heterogeneity, a characteristic that is generally positively reflected in respondents’ visual preferences, as has been demonstrated by other authors too [82,83]. However, in landscapes C and D with higher baseline heterogeneity, the positive effect of smaller farm plot size on the evaluation of the entire landscape scenes was not significant. In these cases, where the heterogeneity of the entire landscape was saturated by the morphology and three-dimensional landscape structures such as trees, woodlands, and hedgerows, the respondents apparently did not appreciate the further increase brought by the smaller plot size. 

This leads us to the interpretation that respondents showed their appreciation by awarding higher beauty scores to scenes where smaller farm plot size harmonized otherwise homogeneous landscapes. The importance of harmony for aesthetic evaluation has been known for centuries. Thomas Aquinas (1224-74) considered harmony, defined by balanced proportions, along with integrity and brightness, to be the three basic determinants of beauty [84]. Landscape preference is strongly dependent on cultural and geographic factors [85,86]. While large-scale landscapes with distant vistas are preferred in some parts of the world [87], in the case of European rural landscapes, people prefer harmonious agricultural landscapes with a balanced ratio of natural and cultural features presented as small-scale landscape patterns [88,89]. 

In addition to the smaller landscape scale, another notable effect of smaller farm plot size is a more varied color effect, determined by patch richness. Smaller plots with alternating crops, with a gradually changing color aspect depending on the current crops and their vegetation phase, are the carrying features of color diversity and contrast in the agricultural landscape. These agricultural parts of the landscape also represent a dynamic mosaic of varying shapes and colors, changing annually at a pace that is traditional and commensurate with human perception. The significance of harmony and color contrast for the aesthetic evaluation was explained in detail by Chevreul [90] as early as 1855, with implications for architecture coming, e.g., from Lynch [91], and landscape applications, e.g., from Kaplan and Kaplan [92]. Research in landscape ecological disciplines [17,53] has shown that functional landscape heterogeneity, which can be facilitated by a smaller-scale plot pattern, plays an equally important role in the conservation of biodiversity in agricultural landscapes as the traditionally recognized habitat patches. In view of this paradigm, our results make a contribution to the ongoing debate on whether people prefer presumably more sustainable landscapes over less sustainable landscapes [16], suggesting that there may be an innate appreciation of agricultural landscapes with a high level of functional landscape heterogeneity. 

The preference for living in a harmonious landscape is also significant in the context of health issues. A society facing an increasing onset of stress-related diseases tends to return to the empirical experience of a positive influence on the health and well-being of visually appealing landscapes. While a large proportion of research in this field deals with the significance of natural landscape features, some studies have also confirmed the effect of diverse small-scale farmland patterns [93,94]. A number of studies, concisely overviewed by Velarde et al. [95], have shown that a positive aesthetic landscape experience, which is enhanced by harmonious relations in the landscape, can evoke feelings of tranquility and can even accelerate healing processes. 

Overall, the preferences described here were manifested very consistently across all respondent groups and throughout the Czech Republic. In comparison with most visual preference studies [56,96], the level of consensus among respondents was exceptionally high. There are only a few notable exceptions. Consistently with the findings of most visual preference studies [97,98], women tended to give higher scores to landscapes throughout our study, including the control landscapes. With increasing age, respondents were far more critical of the high level of consolidation in the least diverse landscapes A and B. As farm plot size increased manifold in agricultural landscapes during the second half of the 20th century, and especially during the 1970s and 1980s [99], respondents over 52 years of age, and especially those over 70 years of age, remember the smaller-scale agricultural landscapes of their childhood and also remember witnessing the enlarging of farm plot size and the ensuing loss of farmland diversity and sustainability. However, the childhood landscapes of younger respondents already had a larger plot size, and this may have affected the lower sensitivity of younger respondents to this phenomenon. Respondents with university-level education were more critical than the rest of the sample toward high levels of consolidation in a landscape. This could be connected with a higher level of understanding of the consequences of extremely low landscape heterogeneity. Similarly, people working or studying in disciplines focused on landscape design or conservation (Group 1) were significantly more critical than other groups of respondents toward all variants of landscapes A and B. This indicates a deeper understanding of the negative consequences of low baseline landscape heterogeneity. 

Finally, our results indicate that even a moderate change in the field size would lead to the perception of these landscapes as more beautiful, and therefore more attractive to live in or to visit. Along with the corresponding findings in landscape ecology on the importance of functional landscape heterogeneity, our results form a basis for agricultural policy development that will ensure the ecological and social sustainability of agricultural landscapes.

## Figures and Tables

**Table 1:** Characteristics of respondents and their categories contained in the questionnaire.

Respondent Characteristics	Categories
Gender	male, female
Age	18–35 years, 36–52 years, 53–69 years, 70 years and over
Education	elementary, high school, university
Place of birth	municipality of birth
Current place of residence	municipality of current residence
Field of occupation/study	professions connected with landscape design and conservation (Group 1)—architecture, landscape and urban planning, ecology, and nature conservation; professions connected with production in the landscape (Group 2)—agriculture and forestry; professions not directly connected to landscape planning, use, or conservation (Group 3)

## Data Availability

The data that support the findings of this study are available upon request from the authors.
